# A novel approach to predict cetuximab‐induced hypersensitivity reaction: detection of drug‐specific IgE on basophils

**DOI:** 10.1002/cam4.658

**Published:** 2016-02-16

**Authors:** Takuya Iwamoto, Akiharu Okamoto, Hajime Ishinaga, Kasumi Shimizu, Alberto A. Gayle, Naoya Arai, Kazuhiko Takeuchi, Masahiro Okuda

**Affiliations:** ^1^ Department of PharmacyMie University HospitalTsuMie514‐8507Japan; ^2^ Department of Otorhinolaryngology‐Head and Neck SurgeryMie University Graduate School of MedicineTsuMie514‐8507Japan; ^3^ Department of Oral and Maxillofacial SurgeryMie University Graduate School of MedicineTsuMie514‐8507Japan; ^4^ Center for Medical and Nursing EducationMie University Graduate School of MedicineTsuMie514‐8507Japan

**Keywords:** Antibody drugs, biomarkers, drug allergy, head and neck cancer

## Abstract

Cetuximab is remarkable for the relatively high rate and severity of hypersensitivity reactions (HR) being reported in the literature. Screening for cetuximab‐specific IgE in serum via immunoassay has been found to be useful in preventing HR; however, these tests are known to have a low positive predictive rate. In an attempt to remedy this, we evaluated the interaction between cetuximab and IgE on basophils for predicting severe cetuximab‐induced HR. Twelve head and neck cancer patients were enrolled in this single‐institution study: four with a history of cetuximab‐induced HR and eight with no such history. Cetuximab‐specific and galactose‐*α*‐1,3‐galactose (*α*‐gal) specific IgEs in serum were measured in vitro using an enzyme‐linked immunosorbent assay (ELISA). IgE‐cetuximab binding on basophils was also analyzed to evaluate the decrease in cetuximab molecules on basophils after dissociation of IgE from FcεRI. The positive predictive value associated with the presence of cetuximab‐ or *α*‐gal‐specific IgE in serum was found to be only 0.67, whereas the negative predictive value was 1.00. On the other hand, in all four patients who developed HR, the cetuximab molecules on basophils were decreased significantly due to the dissociation of IgE from basophils (*P* < 0.05). However, this was not the case in patients who did not develop HR. In conclusion, our results strongly imply that the IgE‐cetuximab interaction on basophils may be key to developing improved methods for predicting severe cetuximab‐induced HR.

## Introduction

Cetuximab, a chimeric mouse–human IgG1 monoclonal antibody, is an epidermal growth factor receptor antagonist that is widely used for the treatment of metastatic colorectal cancer and squamous cell carcinoma of the head and neck 1. Monoclonal antibodies are known to be less toxic than conventional cytotoxic anticancer drugs and are generally well tolerated; however, monoclonal antibodies may sometimes cause severe hypersensitivity reaction (HR). The reported incidence of cetuximab‐associated severe HR (grade 3 or 4) has varied from 1.1% to 5.0% 1, 2, 3, 4, 5, 6, with even higher rates having been reported in Tennessee and North Carolina (18.9% and 25.7%, respectively) 7, 8. In such cases, the clinical symptoms most often associated with severe HR were rapid onset of airway obstruction, hypotension, shock, loss of consciousness, myocardial infarction, and/or cardiac arrest 9, 10. The acute nature and severity of these symptoms 11 are suggestive of a type I allergic reaction mediated by preexisting IgE antibodies cross‐reactive with cetuximab.

Indeed, previous studies have demonstrated a clear link between cetuximab‐induced severe HR and the presence of cross‐reactive IgE antibodies in the serum 12. The IgE antibody has also been shown to be cross‐reactive with the oligosaccharide, galactose‐α‐1,3‐galactose (*α*‐gal), present in the Fab portion of the cetuximab heavy chain 12, 13. And in patients with severe cetuximab‐induced HR, the cross reaction between IgE antibodies and both *α*‐gal and cetuximab have already been observed 12. In this study, anti‐cetuximab IgE antibodies were detected in 17 out of 25 patients who displayed severe HR, whereas in patients without HR, this was only found in one out of 51. This finding suggests that the cetuximab‐specific IgE in serum may be a possible biomarker for predicting severe HR in clinical settings. Furthermore, there have been two large‐scale studies evaluating the presence of cetuximab‐specific IgE in serum. These studies demonstrated that the absence of cetuximab‐specific IgE was definitively associated with HR negative status in almost all cases. However, positive test results were not found to be strongly associated with severe HR (positive predictive value 0.333–0.577) 12, 14. Therefore, a screening test with much higher positive predictive value is clearly needed in order to predict and prevent severe HR due to cetuximab infusion.

Due to their role in the release of inflammatory mediators, basophils have become a useful target for understanding and diagnosing IgE‐mediated allergic reactions 15. CD203c has been identified as a specific surface marker for basophils and mast cells of the hematopoietic lineage 16. It is expressed on resting cells at low levels with a rapid up‐regulation of expression following activation 17. CD203c was also considered to be a more specific marker than CD63 for IgE‐mediated basophil activation 18. Recently, ex vivo exposure to cetuximab was found to induce basophil activation in two out of two patients with an allergy to red meat, in whom *α*‐gal‐specific IgE were detected 19. Considering the relationship between *α*‐gal‐ and cetuximab‐specific IgE, these findings lead us to conclude that a more precise understanding of the pharmacodynamic action of cetuximab on basophils could be useful for predicting cetuximab‐induced severe HR.

The aim of this study is to construct a more effective way of predicting cetuximab‐induced HR in the clinical setting. To accomplish this, we examine differences in the interaction between cetuximab and IgE on basophils in patients with and without cetuximab‐induced HR. This research was designed to contribute to the development and discovery of improved pharmacodynamics‐based methods for the early detection of potentially life‐threatening allergic reactions.

## Materials and Methods

### Study subjects

This study included patients who were admitted to the Mie University Hospital between April 2014 and June 2015. The entry criteria were as follows: diagnosis of head and neck cancer, history of cetuximab‐induced HR within the past 3 months or initiation of cetuximab therapy within the next week. Twelve patients with head and neck cancer were enrolled in this study; characteristics of the patients are shown in Table 1. Two patients had a history of grade 3 allergy during the first cetuximab infusion. Ten patients were started on cetuximab therapy after enrollment; of these, two patients developed grade 1 allergy during the first cetuximab infusion, whereas eight had no allergic reactions. The degree of HR was evaluated according to the Common Terminology Criteria for Adverse Events Version 4.0. All 12 patients received dexamethasone and an H_1_‐receptor antagonist just prior to cetuximab administration. Upon study entry, blood samples were obtained from the patients on two separate days.

**Table 1:** Characteristics of the head and neck cancer patients who participated in this study

No of patients	12
Age (years)	65 (52–89)
Gender (M/F)	9 / 3
Primary tumor state (*n*)	
Pharyngeal cancer	7
Laryngeal cancer	1
Lingual cancer	3
Parotid gland cancer	1
Metastatic disease (*n*)	9
Concomitant radiation therapy (*n*)	8
Premedication (*n*)	
Diphenhydramine	10
Dexamethazone	12
d‐Chlorpheniramine	2
Celecoxib	1
Famotidine	2
History of tumor surgery (*n*)	5
Development of cetuximab‐induced allergic reaction^a^ (*n*)	4 (Grade 3: 2, Grade 1: 2)

a The severity of allergic reaction was evaluated according to the National Cancer Institute Common Terminology Criteria for Adverse Events version 4.0.

This study was conducted in accordance with the Declaration of Helsinki and its amendments. This study's protocol was reviewed and approved by the Ethics Committee of Mie University (certification No. 2717), and written informed consent was obtained from each subject.

### Serum assays for specific IgEs and total IgE detection

Cetuximab‐specific IgE in serum was measured in vitro using an enzyme‐linked immunosorbent assay (ELISA) according to the method reported by Mariotte et al. 14, with minimal modifications. High protein‐binding capacity polystyrene 96‐well plates (Maxisorp Nunc, Thermo Scientific, Yokohama, Japan) were coated with 100 *μ*L of a 0.5 *μ*g/mL cetuximab solution (Erbitux^®^, Merck Serono, Tokyo, Japan) overnight at 4°C. Next, the plates were saturated with 1% human albumin solution for 2 h at 37°C. Duplicate serum samples (diluted 1/10) were added and incubated overnight at 4°C. The binding of IgE antibodies to cetuximab was detected using 500 ng/mL biotinylated goat monoclonal anti‐human‐IgE (Vector laboratories, Burlingame, CA) and allowed to react for 1.5 h at 37°C. Streptavidin‐alkaline phosphatase (Sigma‐Aldrich, Osaka, Japan), 1/2000 dilution, was added, followed by 1 mg/mL paranitrophenyl phosphate solution (PNPP, Interchim, Montluçon, France). Optical density was measured at 415 nm by microplate reader (iMark^™^, Bio‐Rad, Hercules, CA).

As for *α*‐gal‐specific IgE detection, 0.5 *μ*g/mL *α*‐gal ‐*β*‐1,4‐N‐acetylglucosamine‐*β*‐spacer‐biotin (GlycoTech Corporation, Gaithersburg, MD) was incubated in streptavidin‐coated 96‐well plates (Nunc Streptavidin Plate C96 Transparent, Thermo Scientific, Yokohama, Japan) for 1 h at room temperature. The binding of IgE antibodies to *α*‐gal was detected using 100 ng/mL of horseradish peroxidase‐conjugated goat polyclonal anti‐human‐IgE (Bethyl laboratories, Montgomery, TA) and soluble 3,3′, 5, 5′‐ tetramethyl‐benzidine (Roche Diagnostics K.K., Tokyo, Japan). Optical density was measured at 450 nm by microplate reader.

The results were expressed as international units (IU) per milliliter (with 1 IU equivalent to approximately 2.4 ng IgE). Human myeloma IgE (Calibiochem, Billerica, MA) used as standard samples were prepared from 0.2 to 170 IU/mL. Total IgE concentration was also measured according to the rate of patients' serum samples directly binding to the solid phase of Maxisorp Nunc microplate. The limit of detection for cetuximab‐ and *α*‐gal‐specific IgEs and total IgE were 0.2 IU/mL. Calibration curves for cetuximab‐ and *α*‐gal‐specific IgE and total IgE had good linear correlations between respective IgE concentration and absorbance; these correlation coefficients (*r* ^2^) were 0.999 (*n* = 2), 0.999 (*n* = 2), and 0.998–0.999 (*n* = 2), respectively.

### Measurement of CD203c expression on basophils

Measurement of CD203c expression on basophils was carried out within 6 h of blood sampling. Patients' peripheral blood mononuclear cells (PBMCs) were isolated from whole blood anticoagulated with EDTA. The Allergenicity Kit (Beckman Coulter, Fullerton, CA) was used for the quantification of basophil CD203c expression according to the instructions supplied by the manufacturer 20. We used 10 *μ*g/mL cetuximab, anti‐IgE antibody (4 *μ*g/mL) as a positive control, and 0.9% NaCl solution as a negative control. Leukocytes in each sample were then analyzed using a flow cytometer (FACSCanto II, Becton Dickinson Japan, Tokyo, Japan). Basophils were identified by their characteristic forward and side scatter, by the expression of CRTH2, and by the absence of CD3.

### Evaluation of IgE‐ cetuximab binding on basophils

Patients' PBMCs were incubated for 2 min on ice with an LS(+) buffer (10 mmol/L lactic acid, 130 mmol/L NaCl, 5 mmol/L KCl, pH3.9) to dissociate IgE from FcεRI on basophils 21, 22. As a control, patient's PBMCs were incubated for 2 min on ice with an LS(−) buffer (130 mmol/L NaCl, 5 mmol/L KCl, pH3.9). To confirm the dissociation of IgE from FcεRI by acid treatment, LS(+) or LS(−) buffer‐treated basophils were stained with a fluorescein isothiocyanate (FITC)‐conjugated anti‐IgE (Dako, Tokyo, Japan) and FITC‐conjugated anti‐FcεRI antibody (CRA1 or CRA2; Bio Academia, Osaka, Japan) and analyzed using a flow cytometer 23. Subsequently, to confirm IgE‐cetuximab binding on basophils, Alexa Fluor 405‐conjugated cetuximab was used. Alexa Fluor 405‐conjugated cetuximab was prepared by Mix‐n‐stain^TM^ CF ^TM^ 405S antibody labeling kit (Biotium, Hayward, CA). We then evaluated the difference between basophils treated with LS(+) and LS(−) buffers with respect to the fluorescence intensity of Alexa Fluor 405.

### Statistical analysis

The non‐parametric Wilcoxon signed‐rank test was used to assess intergroup differences between two groups in cases where two samples were correlated. We also performed the Spearman rank‐order correlation coefficient to evaluate the linear relationship between two variables. All statistical analyses were carried out using GraphPad Prism 5 (version 5.01; GraphPad Software, CA). The P values were two‐sided and *P* < 0.05 was considered statistically significant.

## Results

### Serum assays for IgE antibody

**Figure 1:**
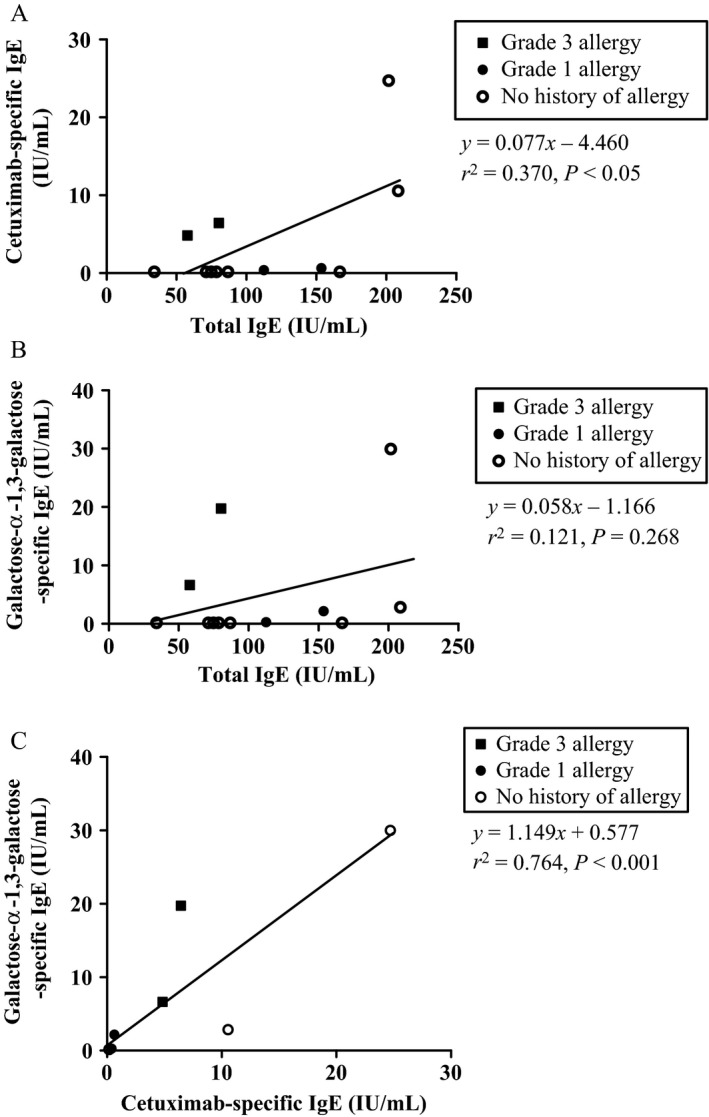
Correlation of cetuximab‐specific IgE, galactose‐*α*‐1,3‐galactose (*α*‐gal)‐specific IgE and total IgE levels in serum. (A) Total IgE in serum versus cetuximab‐spesific IgE in serum. (B) Total IgE in serum versus *α*‐gal‐specific IgE in serum. (C) Cetuximab‐spesific IgE in serum versus *α*‐gal‐specific IgE in serum.

Serum samples found to be positive for cetuximab‐ or *α*‐gal‐specific IgE had antibody titers ranging from 0.2 to 30.0 IU/mL. As for total IgE, antibody titers ranged from 34.2 to 208.4 IU/mL. Statistically significant correlation was observed between the serum levels of total IgE and cetuximab‐specific IgE (*P* < 0.05) (Fig. 1A) as well as cetuximab‐specific IgE and *α*‐gal‐specific IgE (*P* < 0.001) (Fig. 1C). Regarding the predictive value, 67% of patients who tested positive for IgE cross reactive with cetuximab or *α*‐gal were found to have developed HR (positive predictive value: 0.67), whereas 100% of those who did not test positive never experienced HR (negative predictive value: 1.00) (Table 2). The sensitivity and specificity of the assay were 1.00 and 0.75, respectively. (Table 2).

**Table 2:** Association of specific IgE status with allergic reaction

	Allergic reaction (+) (*n *=* *4)	Allergic reaction (−) (*n *=* *8)	Predictive value
Specific IgE (+)	4	2	Positive: 0.67
Specific IgE (−)	0	6	Negative: 1.00
Odds ratio	23.4 (0.893–613)		
	*P* = 0.0606		
Sensitivity	1.00		
Specificity	0.75		

### Basophil activation in response to ex‐vivo cetuximab exposure

**Figure 2:**
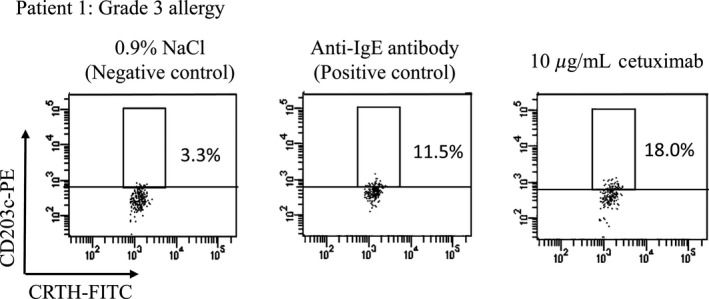
Expression levels of CD203c‐positive basophils following ex‐vivo exposure to 0.9% NaCl (negative control), anti‐IgE antibody (positive control), and 10 *μ*g/mL cetuximab. Peripheral blood mononuclear cells (PBMC) isolated from whole blood of patient 1 (grade 3 allergy) were stained for CD3, CRTH2, and CD203c. Flow cytometer charts for CD3− and CRTH2^+^ cells (basophils) are shown. Up‐regulation of CD203c on basophils was evaluated against a threshold defined as the expression level above which 3.3% of basophils in the negative control column fluorescence.

Basophil activation tests with cetuximab were performed in all 12 patients (Table S1). Positive CD203c basophils were identified by the relatively higher fluorescence intensity of anti‐CD203c antibodies in comparison with that of the top 2–3% of unstimulated control cells (negative control). Ex vivo cetuximab exposure was found to increase activated basophil count in only one patient (Patient 1) who had a history of grade 3 allergy; CD203c‐positive cells (%) and ΔMFI (increase in median fluorescence intensity against negative control exposure) were 18.0% and 135, respectively (Fig. 2). No other patients, including another patient with grade 3 allergy, demonstrated any such increase in basophil activation upon ex vivo exposure to cetuximab.

### Evaluation of IgE‐cetuximab binding on basophils

**Figure 3:**
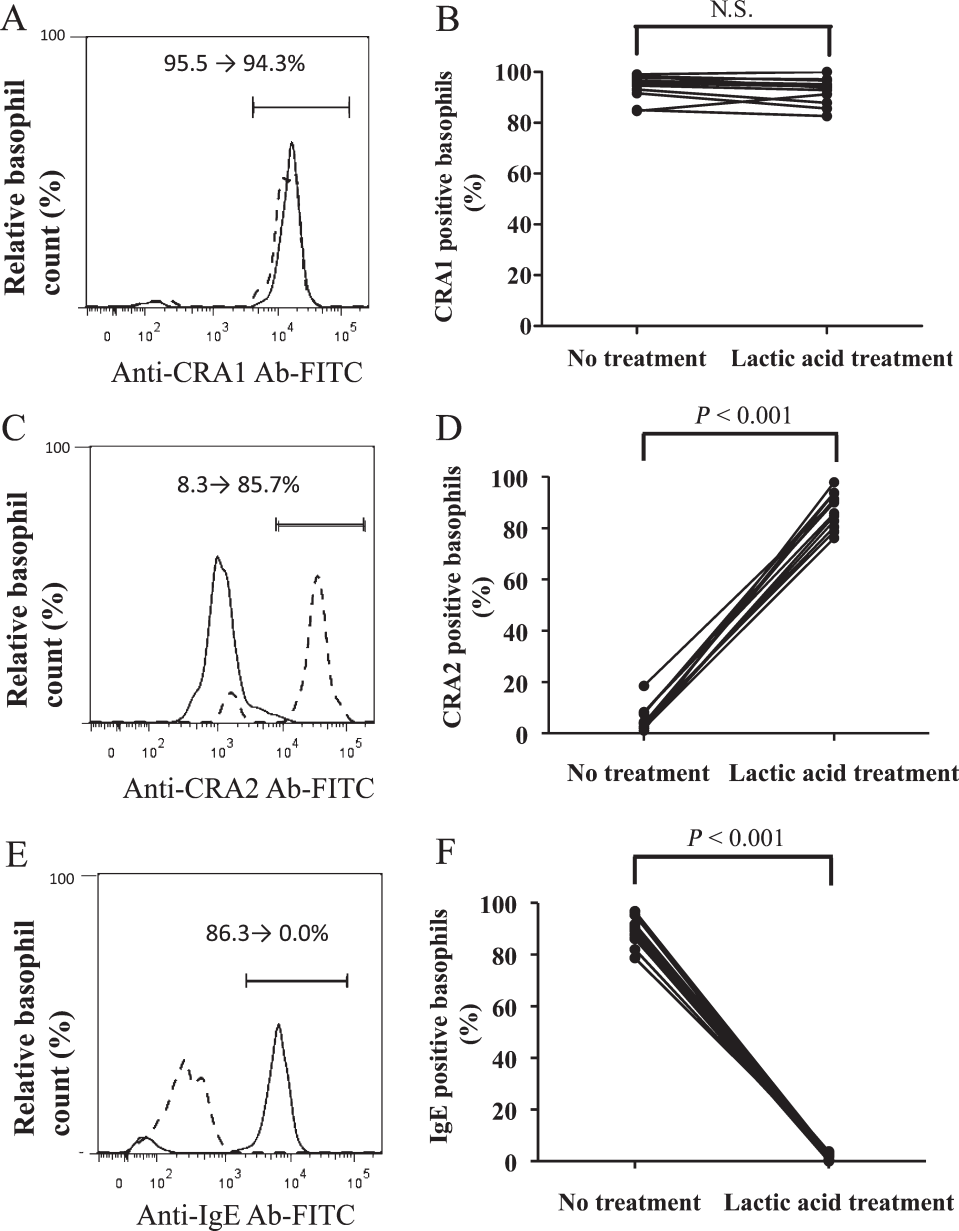
Levels of binding of CRA1, CRA2, and anti‐IgE antibody measured via basophil staining with or without lactic acid treatment by flow cytometric analysis. The levels of CRA1, CRA2, and IgE on basophils in the patient 1 (grade 3 allergy) are shown in (A, C, and E), respectively. In each figure, dashed and solid lines mean with and without lactic acid treatment, respectively. Levels of CRA1, CRA2, IgE on basophils with and without lactic acid treatment were compared in all 12 patients, which are shown in (B, D, and F), respectively. Differences in paired samples were analyzed using the Wilcoxon signed‐rank test.

Flow cytometry analysis was subsequently used to measure the IgE‐cetuximab binding on basophils. This binding was assessed by observing the decrease in fluorescence intensity of Alexa Fluor 405‐conjugated cetuximab affixed to basophils upon IgE dissociation. This dissociation of IgE from basophils was confirmed by staining either with the anti‐FcεRI antibodies, CRA1 and CRA2, or with an anti‐IgE antibody. As already discussed, CRA1 does not bind directly with the IgE‐binding site, whereas CRA2 does.^24^ We were thus able to confirm the successful dissociation of IgE by confirming that lactic acid treatment increased the staining levels of CRA2 (*P* < 0.001) (Fig. 3C and D) and decreased the staining levels of anti‐IgE antibody (*P* < 0.001) (Fig. 3E and F). CRA1 staining levels, on the other hand, remained consistent irrespective of whether lactic acid was applied or not. (Fig. 3A and B).

**Figure 4:**
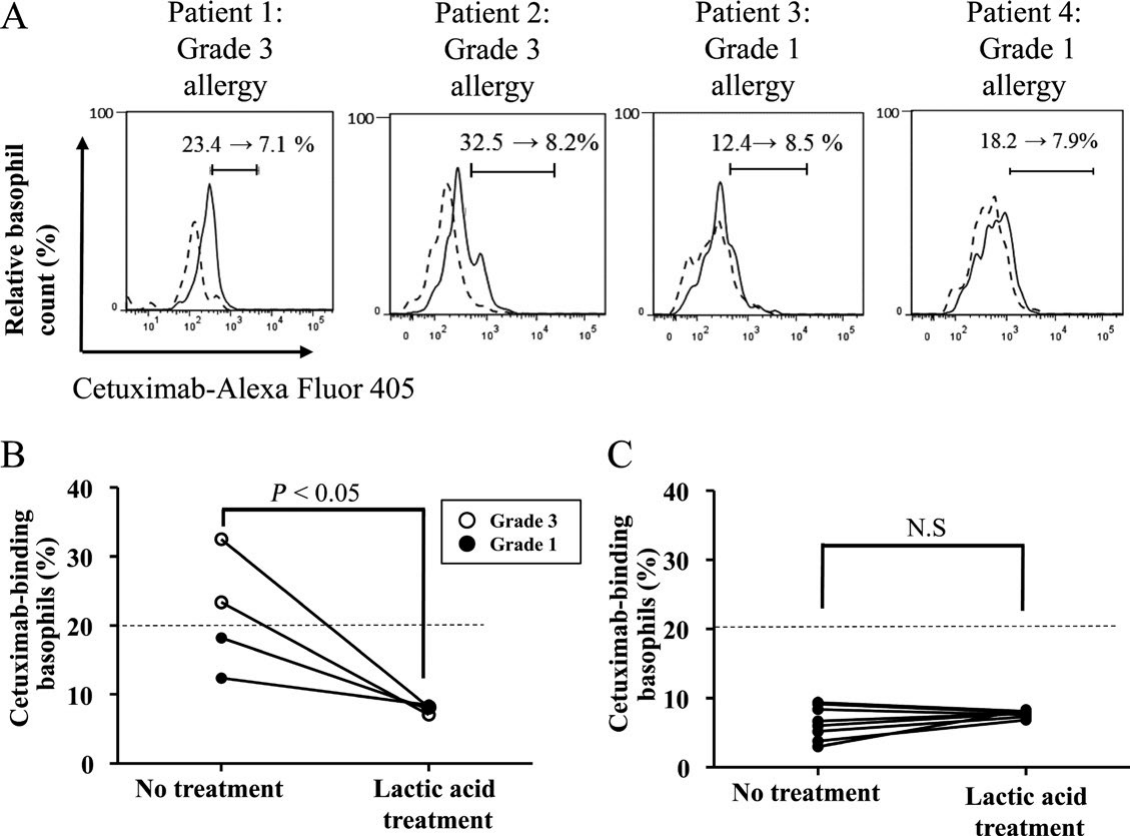
Levels of binding of Alexa Fluor 405‐conjugated ‐cetuximab on basophils were measured via basophil staining with or without lactic acid treatment. (A) Flow cytometer charts in all four patients (patients 1, 2, 3 and 4) with a history of cetuximab‐induced hypersensitivity reactions HR. In each figure, dashed and solid lines mean with and without lactic acid treatment, respectively. (B) Difference in the percentage of cetuximab‐binding basophils with and without lactic acid treatment in all 4 patients with a history of cetuximab‐induced HR. (C) Difference in the percentage of cetuximab‐binding basophils with and without lactic acid treatment in 8 patients without the history of cetuximab‐induced HR. Differences in paired samples were analyzed using the Wilcoxon signed‐rank test.

Figure 4A shows the respective levels of binding of Alexa Fluor 405‐conjugated cetuximab measured via basophil staining with or without lactic acid treatment in 4 HR patients (patients 1, 2, 3, and 4). The staining levels of cetuximab on basophils in HR patients were significantly decreased by lactic acid treatment (*P* < 0.05), especially in patients with grade 3 allergy (Fig. 4A and B), whereas this was not observed in patients with no HR (Fig. 4C).

## Discussion

Several studies have been conducted in order to predict cetuximab‐induced severe HR based on the IgE antibody's recognition of cetuximab's Fab glycosylation site. These studies have demonstrated that the absence of the specific IgE in serum consistently results in negative HR in almost all cases. The same, however, cannot be said for positive test results; hence, the relatively low positive predictive value 12, 14. In our study, we obtained similar results: the positive predictive value associated with the presence of cetuximab‐ or *α*‐gal‐specific IgE was found to be only 0.67, whereas the negative predictive value was 1.00, with a good linear relationship shown between the expression levels of the cetuximab‐ and *α*‐gal‐specific IgEs in all study patients (Fig. 1C).

Interestingly, we found extraordinarily elevated total IgE levels in the serum of the two patients with false positive results for both cetuximab‐ and *α*‐gal‐specific IgEs. This may help to further explain the significant linear relationship observed between the levels of total IgE and cetuximab‐specific IgE observed in this study. One may interpret this result to be a probabilistic binding of serum IgE with the structure of either cetuximab or *α*‐gal in vitro, due to overall higher total IgE levels in these patients. Indeed, other studies have shown that individuals with higher overall IgE levels are likely to have antibodies specific to common allergens, such as, in this case, bee / wasp venom and rapeseed 25. However, these dissociations were not considered to be clinically relevant at the time.

To improve the positive predictive value for cetuximab‐induced severe HR, we focused our efforts on the precise characterization of the interaction between cetuximab and the IgE present at the site of action most closely associated with IgE‐mediated HR, basophils. In one other chimeric monoclonal antibody, rituximab, ex vivo exposure was reported to have induced basophil activation in five patients with HR 26. Although there have been no reports directly examining the relationship between the occurrence of cetuximab‐induced HR and basophil activation tests, cetuximab‐induced basophil activation was observed in two out of two patients with *α*‐gal‐specific IgE and allergies to red meat 19. In another study investigating the relation between mammalian meats‐induced allergy and basophil activation, 10 out of 12 subjects with IgE specific to *α*‐gal had demonstrated clinical evidence of allergy during the mammalian meat challenge, and basophil activation correlated with the appearance of clinical symptoms 27.

In our study, only one of our two grade 3 allergy patients demonstrated increased basophil activation based on CD203c expression due to ex vivo cetuximab exposure. Our other grade 3 allergy patient, however, exhibited no such activation. This latter patient's basophils also exhibited a weak response against the positive control (anti‐IgE antibody), which makes it likely possible that they may have simply been a weak responder to this particular basophil activation test. This contention is supported by previous studies, which demonstrated that nonresponders to the basophil activation test using cetuximab as an antigen may indeed exist 19. Furthermore, in both patients with grade 1 allergy, no basophil activation was detected following ex vivo exposure to cetuximab. We previously demonstrated that one patient with repeated carboplatin administration showed no basophil activation just prior to and after the occurrence of grade 1 allergy 28. Thus, we find it reasonable to conclude that basophil activation tests may not always be applicable for predicting or diagnosing HR, particularly at lower grades.

Next, we tried to evaluate the difference between patients with and without cetuximab‐induced HR in terms of IgE‐cetuximab binding on basophils. In all four patients who developed HR, the staining levels of Alexa Fluor 405‐conjugated cetuximab bound to basophils were found to have been significantly decreased by the dissociation of IgE from basophils (Fig. 4A and B). However, this was not the case in patients who did not develop HR (Fig. 4C). In the two patients who produced false‐positive results via ELISA, we also obtained negative results for cetuximab‐IgE binding on basophils. This is the first evidence of its kind to demonstrate that evaluation of cetuximab‐IgE binding on basophils might be a more reliable predictor of HR in terms of reducing false‐positive results.

The timing of blood sampling may have influenced the ex vivo basophil tests in the two patients with grade 3 allergy. In these patients, the tests were performed more than 44 days after final cetuximab administration. The elimination half‐life of cetuximab is approximately 5 days. Cetuximab was therefore presumed to have been almost completely eliminated from circulation in such cases. Consequently, we expect that prior administration of cetuximab had minimal bearing on these results. In addition, IgE‐cetuximab binding on basophils was found to have decreased due to dissociation of IgE from basophils one day prior to (12.4–8.5%) and 7 days after the occurrence of hypersensitivity reaction (12.2–8.1%). This result supports the idea that pre‐exposure to cetuximab had negligible influence on the results reported in this study.

Recently, it has been reported that the basophil activation test may be much more sensitive than the respective IgE detection methods offered by ELISA or ImmunoCAP, at least in the case of recombinant allergens such as those found in wasp venom 29. This supports our results that interaction between cetuximab and specific IgE on basophil rather than detection of specific IgE in serum correctly reflect the clinical onset of allergic reaction. Furthermore, evaluation of specific IgE on basophils via flow cytometer has an advantage for clinical application in that the measurement times are usually less than 3 h, whereas detection of antigen‐specific IgE via conventional ELISA usually takes 2–3 days 14. It has been known that, in addition to high‐affinity FcεRI, human basophils also express low‐affinity IgG receptors, Fc*γ*RIIA and Fc*γ*RIIB as well as minute amounts of the low‐affinity IgG receptor, Fc*γ*RIIIB 30. Although cetuximab, an IgG1 monoclonal antibody, has a possibility to bind to these Fc*γ* receptors on basophils, the binding rate seems relatively low because of its low affinity and/or low expression on basophil.

Although virtually no premedication can be shown to definitively prevent HR due to cetuximab, the administration of corticosteroids before infusion of cetuximab has been shown to be effective in limiting the occurrence of severe HR [31]. Accordingly, an improved understanding of the pharmacodynamic property of cetuximab on basophils may be helpful for identifying and developing appropriate treatment plans for high‐risk patients requiring pretreatment with corticosteroids.

In conclusion, our results strongly imply that quantification of the IgE‐cetuximab interaction on basophils is an attractive method for identification of high‐risk patients with severe HR. Although this study had a small sample size drawn from a single institute, we believe that these results may be instrumental in understanding the difference between the reactivity of serum IgE and IgE binding on basophils. We anticipate this to be a key first step toward the development of more highly sensitive tests for predicting cetuximab‐induced severe HR as well as personalized premedication regimens for cetuximab administration. Furthermore, we feel our method for evaluating drug‐IgE interaction on basophils could be adapted and applied for the evaluation of other monoclonal antibody drugs. Ultimately, we feel this work should be of considerable interest to anyone interested in drug‐target molecule interactions, as well as healthcare workers and researchers concerned with the safe management of anti‐cancer antibody treatment.

## Conflict of Interest

No conflicts of interests were declared by the other authors.

## References

[1] Company B‐MS . ERBITUX® (cetuximab) injection. 2013.

[2] Cunningham, D. , Y. Humblet , S. Siena , D. Khayat , H. Bleiberg , A. Santoro , et al. 2004 Cetuximab monotherapy and cetuximab plus irinotecan in irinotecan‐refractory metastatic colorectal cancer. N Engl J Med. 351:337–45. 15269313 10.1056/NEJMoa033025

[3] Bonner, J. A. , P. M. Harari , J. Giralt , N. Azarnia , D. M. Shin , R. B. Cohen , et al. 2006 Radiotherapy plus cetuximab for squamous‐cell carcinoma of the head and neck. N Engl J Med. 354:567–78. 16467544 10.1056/NEJMoa053422

[4] Vermorken, J. B. , R. Mesia , F. Rivera , E. Remenar , A. Kawecki , S. Rottey , et al. 2008 Platinum‐based chemotherapy plus cetuximab in head and neck cancer. N Engl J Med. 359:1116–27. 18784101 10.1056/NEJMoa0802656

[5] Pirker, R. , J. R. Pereira , A. Szczesna , J. von Pawel , M. Krzakowski , R. Ramlau , et al. 2009 Cetuximab plus chemotherapy in patients with advanced non‐small‐cell lung cancer (FLEX): an open‐label randomised phase III trial. Lancet. 373:1525–31. 19410716 10.1016/S0140-6736(09)60569-9

[6] Galvao, V. R. . and M. C., Castells Hypersensitivity to biological agents‐updated diagnosis, management, and treatment. J Allergy Clin Immunol Pract. 2015; 3: 175–85; quiz 86. 25754718 10.1016/j.jaip.2014.12.006

[7] O'Neil, B. H. , R. Allen , D. R. Spigel , T. E. Stinchcombe , D. T. Moore , J. D. Berlin , et al. 2007 High incidence of cetuximab‐related infusion reactions in Tennessee and North Carolina and the association with atopic history. J Clin Oncol. 25:3644–8. 17704414 10.1200/JCO.2007.11.7812

[8] Keating, K. , C. Walko , B. Stephenson , B. H. O'Neil , and J. Weiss . 2014 Incidence of cetuximab‐related infusion reactions in oncology patients treated at the University of North Carolina Cancer Hospital. J Oncol Pharm Pract. 20:409–16. 24243920 10.1177/1078155213510542

[9] Dupont, B. , D. Mariotte , C. Moldovan , J. M. Grellard , M. C. Vergnaud , D. Laroche , et al. 2014 Case Report About Fatal or Near‐Fatal Hypersensitivity Reactions to Cetuximab: Anticetuximab IgE as a Valuable Screening Test. Clin Med Insights Oncol. 8:91–4. 25089092 10.4137/CMO.S13897 PMC4116358

[10] Maier, S. , C. H. Chung , M. Morse , T. Platts‐Mills , L. Townes , P. Mukhopadhyay , et al. 2015 A retrospective analysis of cross‐reacting cetuximab IgE antibody and its association with severe infusion reactions. Cancer medicine. 4:36–42. 25296628 10.1002/cam4.333 PMC4312116

[11] Sampson, H. A. 2003 Anaphylaxis and emergency treatment. Pediatrics. 111:1601–8. 12777599

[12] Chung, C. H. , B. Mirakhur , E. Chan , Q. T. Le , J. Berlin , M. Morse , et al. 2008 Cetuximab‐induced anaphylaxis and IgE specific for galactose‐alpha‐1,3‐galactose. N Engl J Med. 358:1109–17. 18337601 10.1056/NEJMoa074943 PMC2361129

[13] Platts‐Mills, T. A. , A. J. Schuyler , A. Tripathi , and S. P. Commins . 2015 Anaphylaxis to the carbohydrate side chain alpha‐gal. Immunol Allergy Clin North Am. 35:247–60. 25841549 10.1016/j.iac.2015.01.009 PMC4617526

[14] Mariotte, D. , B. Dupont , R. Gervais , M. P. Galais , D. Laroche , A. Tranchant , et al. 2011 Anti‐cetuximab IgE ELISA for identification of patients at a high risk of cetuximab‐induced anaphylaxis. MAbs. 3:396–401. 21654207 10.4161/mabs.3.4.16293 PMC3218536

[15] Boumiza, R. , A. L. Debard , and G. Monneret . 2005 The basophil activation test by flow cytometry: recent developments in clinical studies, standardization and emerging perspectives. Clin Mol Allergy. 3:9. 15989690 10.1186/1476-7961-3-9 PMC1190199

[16] MacGlashan, D. Jr . 2010 Expression of CD203c and CD63 in human basophils: relationship to differential regulation of piecemeal and anaphylactic degranulation processes. Clin Exp Allergy. 40:1365–77. 20633031 10.1111/j.1365-2222.2010.03572.x PMC2927965

[17] Hennersdorf, F. , S. Florian , A. Jakob , K. Baumgartner , K. Sonneck , A. Nordheim , et al. 2005 Identification of CD13, CD107a, and CD164 as novel basophil‐activation markers and dissection of two response patterns in time kinetics of IgE‐dependent upregulation. Cell Res. 15:325–35. 15916720 10.1038/sj.cr.7290301

[18] Chirumbolo, S. , A. Vella , R. Ortolani , M. De Gironcoli , P. Solero , G. Tridente , et al. 2008 Differential response of human basophil activation markers: a multi‐parameter flow cytometry approach. Clin Mol Allergy. 6:12. 18925959 10.1186/1476-7961-6-12 PMC2584049

[19] Michel, S. , K. Scherer , I. A. Heijnen , and A. J. Bircher . 2014 Skin prick test and basophil reactivity to cetuximab in patients with IgE to alpha‐gal and allergy to red meat. Allergy. 69:403–5. 24372137 10.1111/all.12344

[20] Nagao, M. , Y. Hiraguchi , K. Hosoki , R. Tokuda , T. Usui , S. Masuda , et al. 2008 Allergen‐induced basophil CD203c expression as a biomarker for rush immunotherapy in patients with Japanese cedar pollinosis. Int Arch Allergy Immunol. 146(Suppl 1):47–53. 18504407 10.1159/000126061

[21] Tanaka, A. , T. Tanaka , H. Suzuki , K. Ishii , Y. Kameyoshi , and M. Hide . 2006 Semi‐purification of the immunoglobulin E‐sweat antigen acting on mast cells and basophils in atopic dermatitis. Exp Dermatol. 15:283–90. 16512875 10.1111/j.0906-6705.2006.00404.x

[22] Suzuki, H. , Y. Yanase , T. Tsutsui , K. Ishii , T. Hiragun , and M. Hide . 2008 Applying surface plasmon resonance to monitor the IgE‐mediated activation of human basophils. Allergol Int. 57:347–58. 18797180 10.2332/allergolint.O-07-506

[23] Iwamoto, T. , H. Hirai , N. Yamaguchi , N. Kobayashi , H. Sugimoto , T. Tabata , et al. 2014 Carboplatin‐induced severe hypersensitivity reaction: role of IgE‐dependent basophil activation and FcepsilonRI. Cancer Sci. 105:1472–9. 25230301 10.1111/cas.12538 PMC4462369

[24] Takai, T. , K. Takahashi , M. Akagawa‐Chihara , M. Fukada , T. Yuuki , I. Shibuya , et al. 2001 Production of humanized antibody against human high‐affinity IgE receptor in a serum‐free culture of CHO cells, and purification of the Fab fragments. Bioscience, biotechnology, and biochemistry. 65:1082–9. 10.1271/bbb.65.1082 11440121

[25] Sturm, G. J. , C. Schuster , B. Kranzelbinder , M. Wiednig , A. Groselj‐Strele , and W. Aberer . 2009 Asymptomatic sensitization to hymenoptera venom is related to total immunoglobulin E levels. Int Arch Allergy Immunol. 148:261–4. 18849617 10.1159/000161586

[26] Piva, E. , F. Chieco‐Bianchi , V. Krajcar , S. Aversa , and M. Plebani . 2012 Adverse reactions in patients with B‐cell lymphomas during combined treatment with rituximab: In vitro evaluation of rituximab hypersensitivity by basophil activation test. Am J Hematol. 87:E130–1. 22987289 10.1002/ajh.23329

[27] Commins, S. P. , H. R. James , W. Stevens , S. L. Pochan , M. H. Land , C. King , et al. 2014 Delayed clinical and ex vivo response to mammalian meat in patients with IgE to galactose‐alpha‐1,3‐galactose. J Allergy Clin Immunol. 134:108–15. 24656556 10.1016/j.jaci.2014.01.024 PMC4125475

[28] Iwamoto, T. , A. Yuta , T. Tabata , H. Sugimoto , E. C. Gabazza , H. Hirai , et al. 2012 Evaluation of basophil CD203c as a predictor of carboplatin‐related hypersensitivity reaction in patients with gynecologic cancer. Biol Pharm Bull. 35:1487–95. 22975499 10.1248/bpb.b12-00150

[29] Balzer, L. , D. Pennino , S. Blank , H. Seismann , U. Darsow , M. Schnedler , et al. 2014 Basophil activation test using recombinant allergens: highly specific diagnostic method complementing routine tests in wasp venom allergy. PLoS One. 9:e108619. 25329342 10.1371/journal.pone.0108619 PMC4201461

[30] Cassard, L. , F. Jonsson , S. Arnaud , and M. Daeron . 2012 Fcgamma receptors inhibit mouse and human basophil activation. Journal of immunology. 189:2995–3006. 10.4049/jimmunol.1200968 22908332

[31] Siena, S. , R, G.‐J. , A. Adenis , J. Thaler , P. Preusser , E. A. Aguilar , et al. Reduced incidence of infusion‐related reactions in metastatic colorectal cancer during treatment with cetuximab plus irinotecan with combined corticosteroid and antihistamine premedication. Cancer. 2010; 116: 1827–37. 20143444 10.1002/cncr.24945

