# Supplementary material for: A novel approach to predict cetuximab‐induced hypersensitivity reaction: detection of drug‐specific IgE on basophils
Source: Cancer Med. 2016 Feb 16;5(6):1004–12. doi: 10.1002/cam4.658 (PMC4924357; doi:10.1002/cam4.658)
Supplement: Supplementary file 1 [file CAM4-5-1004-s001.docx]

Table S1 CD203c expression on basophils in response to cetuximab exposure in the study patients.

| Patient  No. | Allergic reaction  Grade ^†^ | Negative control ^‡^ |  | Positive  control ^§^ | |  | Cetuximab  exposure | |
| --- | --- | --- | --- | --- | --- | --- | --- | --- |
|  |  | CD203c positive  cells (%) |  | CD203c positive  cells (%) | ⊿MFI ^d^ |  | CD203c positive cells (%) | ⊿MFI ^¶^ |
| 1 | 3 | 3.3 |  | 11.5 | 147 |  | 18.0 | 135 |
| 2 | 3 | 1.7 |  | 5.6 | 190 |  | 0.5 | 6 |
| 3 | 1 | 1.9 |  | 76.2 | 871 |  | 1.9 | 26 |
| 4 | 1 | 2.0 |  | 30.7 | 47 |  | 1.5 | 0 |
| 5 | 0 | 2.2 |  | 29.1 | 210 |  | 1.0 | 12 |
| 6 | 0 | 2.1 |  | 28.2 | 401 |  | 1.0 | 0 |
| 7 | 0 | 1.6 |  | 88.0 | 577 |  | 4.3 | 4 |
| 8 | 0 | 1.1 |  | 60.6 | 261 |  | 5.9 | 13 |
| 9 | 0 | 2.5 |  | 9.7 | 117 |  | 2.4 | 9 |
| 10 | 0 | 1.9 |  | 32.2 | 282 |  | 2.5 | 17 |
| 11 | 0 | 2.1 |  | 10.8 | 128 |  | 2.0 | 41 |
| 12 | 0 | 1.5 |  | 59.6 | 505 |  | 0.0 | 6 |

^†^ The severity of anaphylaxis was evaluated using the National Cancer Institute Common Terminology Criteria for Adverse Events version 4.0.

^‡^ Isolated basophils were exposed to 0.9% NaCl.

^§^ Isolated basophils were exposed to specific IgE.

^¶^ Increase in median fluorescence intensity against negative control.
